# Clinical Observation of Allergic Conjunctival Diseases with Portable and Recordable Slit-Lamp Device

**DOI:** 10.3390/diagnostics11030535

**Published:** 2021-03-17

**Authors:** Hiroyuki Yazu, Eisuke Shimizu, Shinri Sato, Naohiko Aketa, Taiichiro Katayama, Ryota Yokoiwa, Yasunori Sato, Kazumi Fukagawa, Yoko Ogawa, Kazuo Tsubota, Hiroshi Fujishima

**Affiliations:** 1 Department of Ophthalmology, Tsurumi University School of Dental Medicine, Kanagawa 230-0063, Japan; fujishima117@gmail.com; 2 Department of Ophthalmology, Keio University School of Medicine, Tokyo 160-0016, Japan; ophthalmolog1st.acek39@keio.jp (E.S.); shinri.sato259@gmail.com (S.S.); nao.nao.pao.pao@gmail.com (N.A.); taiichiro.katayama@gmail.com (T.K.); fukakazu0706@gmail.com (K.F.); yoko.z7@keio.jp (Y.O.); tsubota@z3.keio.jp (K.T.); 3 OUI Inc., Tokyo 160-0022, Japan; ryota.yokoiwa@gmail.com; 4 Department of Preventive Medicine and Public Health, Biostatistics at Clinical and Translational Research Center, Keio University School of Medicine, Tokyo 160-0016, Japan; yasunori.sato@keio.jp; 5 Ryogoku Eye Clinic, Tokyo 130-0026, Japan

**Keywords:** allergic conjunctivital diseases, portable, recordable, conventional slit-lamp microscope, smart eye camera

## Abstract

Background: The incidence of allergic conjunctival diseases (ACDs) is gradually increasing worldwide. Both ophthalmologists and non-ophthalmologists prescribe eye drops to treat ACDs; however, there are many cases which are treated without sufficient examination and diagnosis of the eyes. We have invented a portable, recordable, and smartphone-attachable slit-lamp device—Smart Eye Camera (SEC). The purpose of this study was to compare the diagnostic abilities of ACDs between the SEC and the conventional, non-portable slit-lamp microscope. Methods: This prospective observational study included 32 eyes of 17 Japanese patients (mean age: 21.5 ± 14.8 years; range: 11–51 years; female: 5). The severity of 10 objective signs in the palpebral conjunctiva, bulbar conjunctiva, limbus, and cornea were scored on a grading scale of 0 to 4 (0 = normal; 1+ = mild; 2+ = moderate; 3+ = severe), respectively. First, the conventional slit-lamp microscope was used to examine the grade of the ACDs. Second, another ophthalmologist filmed the eyes using the SEC and two other ophthalmologists evaluated the grades on another day. The correlation and inter-rater reproducibility in total scores among the two devices were determined. Results: Total scores of clinical signs, evaluated by the two approaches, correlated significantly (both eyes: r = 0.918 (95% CI: 0.839 to 0.959; *p* < 0.001)), with substantial inter-rater agreement (weighted *κ* value = 0.631 (95% CI: 0.601 to 0.661; *p* < 0.001)). Conclusions: The SEC is as reliable as the conventional non-portable slit-lamp microscope for assessing ACDs.

## 1. Introduction

The number of patients with allergic conjunctival diseases (ACDs) is increasing worldwide [1,2]. ACDs can be divided into five types according to the presence of proliferative changes, atopic dermatitis (AD), and the presence/absence of mechanical irritation (i.e., atopic keratoconjunctivitis (AKC), vernal keratoconjunctivitis (VKC), giant papillary conjunctivitis (GPC), seasonal allergic conjunctivitis (SAC), and perennial allergic conjunctivitis (PAC)) [3]. AKC and VKC are classified as the severe types of ACDs, because they can cause keratopathy [4,5], such as a shield ulcer or corneal plaque leading to visual morbidity in some cases [6,7,8]. Moreover, these diseases are particularly likely to occur in young people [9,10,11]; therefore, early diagnosis and accurate diagnosis are important. However, some patients are difficult to examine due to their poor conditions of the eyelid (e.g., lid swelling and hard skin), or secondary to difficulty with lid opening or closure [12]. Because GPC is often seen in contact lens wearers [13,14], it can occur at any age; it often improves when once contact lens wearing is stopped. However, some patients refuse to stop wearing contact lenses; thus, it is important to ensure that patients with GPC understand their diagnosis through showing them images. Meanwhile, SAC and PAC are the most common allergic disorders and can occur at any age. Cedar pollen and house dust are major allergens of SAC and PAC in Japan [1]. Most patients complain of ocular itching as a primary symptom, and also suffer from other signs such as conjunctival redness, eyelid swelling, tearing, and discharge [15]. Because SAC and PAC are less severe than other ACDs [16], eye drops are often prescribed without an accurate eye examination in non-ophthalmologic internal medicine, otorhinology or pediatrics [17]. However, steroid eye drops may also be included in the treatment. Even if the risk of side effects (for example, cataracts and glaucoma) [18,19,20] due to the use of temporary steroids is low, it is not preferable to prescribe steroid eye drops without an accurate diagnosis by an ophthalmologist. Side effects can happen in some cases where the diagnosis was originally different and infection occurred, or the intraocular pressure continued to be high [18,20]. This can often happen in depopulated medical areas or in areas where there are few ophthalmologists. Therefore, it is necessary to construct a device capable of accurately recording an image and a system capable of easily consulting with an ophthalmologist in the case of diagnosis by a non-ophthalmologist. 

To overcome this problem, we have invented a portable and recordable slit-lamp device, the “Smart Eye Camera” (SEC), which was reported both in an animal model [21] and in humans [22,23]. This device has a recording function using the smartphone camera and can convert the smartphone light source to the light needed for the ophthalmology diagnosis. In the present study, we evaluated and compared the effectiveness and diagnostic performance for ACD diagnosis between SEC and the conventional slit lamp microscope.

## 2. Materials and Methods

### 2.1. Ethics Approval

The study was conducted according to the guidelines of the Declaration of Helsinki, and the study protocols were approved by the Institutional Review Board of Tsurumi University School of Dental Medicine, Kanagawa, Japan (IRB No. 1634, 15 March 2019). Written informed consent was obtained from all patients via an agreement document.

### 2.2. Study Design

**Figure 1:**
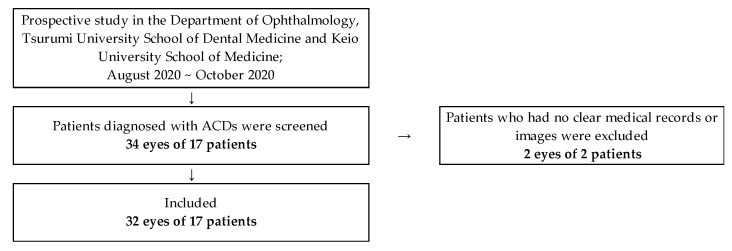
Study flowchart.

In this prospective study, 34 eyes of 17 individuals (12 males and 5 females) were screened. The mean age of patients was 21.5 ± 14.8 years. We screened the patients from August to October 2020. The patients who matched our inclusion criteria were recruited for the study. The inclusion criteria were as follows: (1) Japanese men and women (above 10 years of age), (2) patients who underwent slit-lamp examination with both the conventional slit-lamp microscope and SEC, and (3) no obvious ocular surface infections and/or inflammations. Patients who had at least one of the following were excluded: (1) ocular surgical intervention within 3 months prior to study and/or a history of refractive surgery within 6 months prior to study, (2) received any topical ophthalmic preparations, (3) unclear or lack of data, or (4) did not provide written informed consent. Sample size calculation yielded a required sample size of 17 eyes (details are provided in the Statistical and data analysis section). As a result, two eyes were excluded; we enrolled 32 eyes from 17 Japanese patients between August and October 2020 (Figure 1).

### 2.3. Conventional Non-Portable Slit-Lamp Microscope and SEC Examination

**Figure 2:**
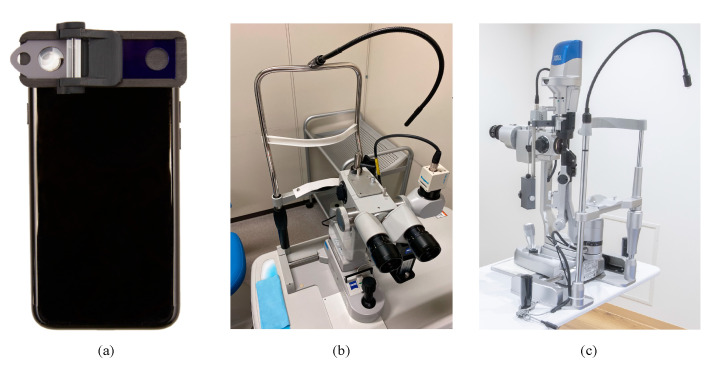
Photographs of the SEC and the conventional slit-lamp microscopes. (**a**) The SEC, (**b**) SL130, and (**c**) 700GL.

We used the SL130 (Carl Zeiss AG, Oberkochen, Germany), and 700GL (Takagi, Nagano, Japan) as the conventional, non-portable slit-lamp microscopes. Each anterior segment image was recorded by the external camera installed in the slit-lamp microscopes (THD-23FHD; IKEGAMI TSUSHINKI CO., LTD., Tokyo, Japan., and MCC-500MD; Sony Business Solutions Corporation, Tokyo, Japan), which was then converted to a JPEG file. The resolution of the photographs was 1920 × 1200 pixels per inch. For a comparison, the SEC (OUI Inc., Tokyo Japan) was selected as a portable and recordable device. SEC is a smartphone attachment that attaches to the smartphone’s light source and camera lens (Figure 2). It has been approved as a medical device in Japan (Japan Medical Device registration number: 13B2X10198030101). The SEC irradiates a blue light at a wavelength of 488 nm when an acrylic resin blue filter (PGZ 302K 302, Kuraray Co., LTD., Tokyo, Japan) is placed above the light source of the smartphone. Moreover, a convex macro lens (focal length, 20 mm; magnification, ×20) is placed above the camera to adjust the focus toward the anterior segment of the eye. The frame was manufactured from polyamide 12 on a 3D printer (Multi Jet Fusion 3D Model 4210; Hewlett-Packard Company, Palo Alto, CA, USA). In this study, the iPhone 7 or 8 (Apple Inc., Cupertino, CA, USA) was used. The resolution of the video was set to 1080 p and 30 frames per second, which is equivalent to 2.1 megapixels (2,073,600 pixels) per file.

### 2.4. Evaluation of Allergic Conjunctival Diseases by the Conventional Slit-Lamp Microscope and the SEC

The recruited cases were examined using both the conventional slit-lamp microscope and SEC. To minimize selection bias in terms of the order of the instrument used, we randomized all cases using a table of random digits. Ten objective signs were assessed using 4 grades (0 = normal; 1+ = mild; 2+ = moderate; 3+ = severe), as in previous reports [3,12,24,25], with palpebral conjunctiva (hyperemia, swelling, follicles, papillae, and giant papillae), bulbar conjunctiva (hyperemia and chemosis), limbus (Trantas dot and swelling), and corneal epithelial disorder. These criteria are summarized in Table S1. First, two ophthalmologists (Hiroyuki Yazu, and Eisuke Shimizu) used the conventional slit-lamp microscope to examine the grade of ACDs. Second, another ophthalmologist (Taiichiro Katayama) used the SEC to record a video of the eyes. These examinations were almost performed under the ordinary light environment in the ophthalmology department (the room was darkened during using fluorescent staining). Third, the images were documented in the medical records after filming. Finally, on another day, ACD grading was performed by two other ophthalmologists (Kazumi Fukagawa, and Hiroshi Fujishima) who were allergy specialists blinded to the patients’ information. When the diagnosis was different among the ophthalmologists, final diagnosis was decided by the majority consensus.

### 2.5. Statistical and Data Analysis

All data were analyzed using Prism software (ver. 6.04 for Mac; GraphPad Software Inc., San Diego, CA, USA) and SPSS software (IBM SPSS statistics ver. 25; IBM Corp, New York, NY, USA). The sample size calculation was performed based on the previous data [24]. The difference between two independent means and correlation between the groups were used to calculate an effect size of 0.85, statistical power of 0.95, and significance level of 0.05. A total sample size of 17 was determined to be adequate. A paired T-test was performed to compare the scores of ACDs differences between the conventional slit-lamp microscope and the SEC. Spearman’s correlation coefficient was used to evaluate the correlation between the severity of 10 objective signs in ACDs obtained using the conventional slit-lamp microscope and SEC. To assess the reproducibility of the ACD grading by the two devices, weighted kappa statistics were conducted. Data were presented as adjusted means ± 95% confidence intervals (CI), ±standard deviation (SD), or ranges. *p*-value < 0.05 was considered to indicate statistical significance.

## 3. Results

### 3.1. Demographics of the Subjects

In the current study, two cases were PAC, nine cases were SAC, one case was GPC, three cases were AKC, and 2 cases were VKC (Table 1). There was no significant difference among two devices in scores of objective signs; the mean severity of the ACD grading in both eyes was 6.6 ± 4.8 based on the evaluation by the conventional slit-lamp microscope and 7.5 ± 3.6 by the SEC (*p* = 0.43, Table 1). 

**Figure 3:**
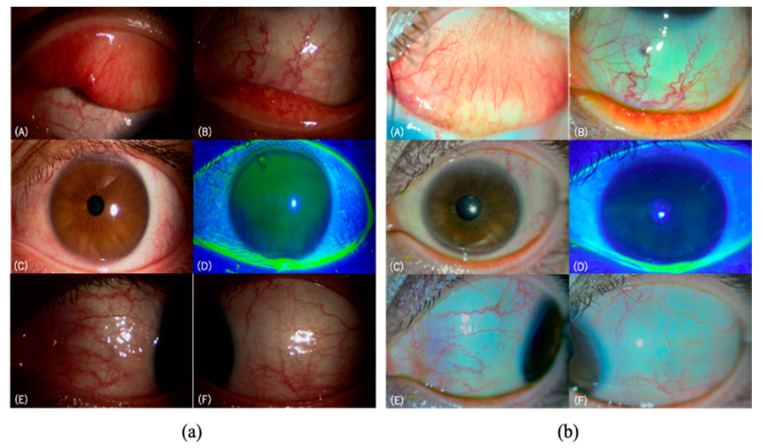
Representative images taken by the two devices; (**a**) the conventional slit-lamp microscope (700GL), and (**b**) the SEC. A 11-year-old female patient was diagnosed with SAC caused by orchard grass. (**A**,**B**) show the palpebral conjunctiva with dilatation of many vessels, localized edema, and a few papillae. There are no follicles or giant papillae. (**C**,**D**) show the limbus and the cornea without swelling, Trantas dot, or epithelial damage. (**E**,**F**) show the bulbar conjunctiva with dilatation of many vessels, and localized edema.

Representative photos taken with the conventional slit-lamp microscope and the SEC are shown in Figure 3.

### 3.2. Correlation of ACD Grading Evaluation by the Two Devices

The total scores of clinical signs by the conventional slit-lamp microscope and the SEC showed a significantly strong correlation in the right eye (r = 0.95 (95% CI: 0.86 to 0.98; *p* < 0.001); Table S2), left eye (r = 0.88 (95% CI: 0.69 to 0.96; *p* < 0.001); Table S2), and both eyes (r = 0.92 (95% CI: 0.84 to 0.96; *p* < 0.001); Table S2). Three of the clinical signs (giant papillae in palpebral conjunctiva, swelling and Trantas dot in limbus) were not observed by both devices in this study; they were scored 0 point in all patients.

### 3.3. Reproducibility of the ACD Grading Evaluated by the Two Devices

Among the two devices, a substantial kappa value was observed in the right eye (Kappa = 0.73 (95% CI: 0.67 to 0.78; *p* < 0.001); Table 2), and both eyes (Kappa = 0.63 (95% CI: 0.60 to 0.66; *p* < 0.001); Table 2), whereas a moderate kappa value was observed in the left eye (Kappa = 0.45 (95% CI: 0.39 to 0.51; *p* < 0.001); Table 2).

## 4. Discussion

Here, we evaluated the diagnostic accuracy and the performance of the SEC in clinical observation of ACDs compared with the conventional slit-lamp microscope. When comparing the severity of objective signs grading between the conventional slit-lamp microscope and the SEC, there was a significantly strong correlation (Table S2). In addition, using weighted kappa statistics to investigate ACD grading showed a substantial kappa value, suggesting that the images got by both devices were substantially reproducible (Table 2). These results suggest that the performance of the SEC is comparable to that of the conventional slit-lamp microscope in the evaluation of ACDs.

A slit-lamp microscope is an indispensable medical instrument in ophthalmology [26]. However, most conventional slit-lamp microscopes are not portable, and patients are required to visit the medical center for ophthalmologic examinations. Therefore, some patients have less opportunity to undergo ophthalmology examinations, and non-ophthalmologists often prescribe medical eye care kits containing eye drops without an accurate diagnosis. There are also handheld microscopy systems, but these do not record. To record images of the anterior segment, an external camera is required, but this requires modification of the equipment. In addition, conventional microscopes are large and heavy because they are operated with one hand, making it difficult to use them outside of ophthalmology clinics, which complicates the verification of conventional non-portable microscopes and portable slit-lamp microscopes. Therefore, it could be possible to solve these problems using the SEC we invented. At present, no study has investigated the ACD evaluation with a portable and recordable slit-lamp device like the SEC. Recently, devices capable of recording anterior segment images have rarely been available, although similar techniques have been reported previously [27,28,29]. However, the light source of the smartphone cannot release blue light for checking epithelial disorders by using fluorescein staining. As shown in Figure 3, it can be noted that, for example, bulbar conjunctival hyperemia and chemosis can be underestimated by SEC due to light intensity. In general, existing slit-lamp microscopes require accurate diagnosis on the spot; however, the accuracy of diagnosis is improved by looking back at the still image. In contrast, the SEC can look back in video (i.e., continuous images). Thus, doctors can provide accurate diagnosis and clear explanations to patients, which may result in improved compliance with the appropriate treatment and a hospital visit.

ACDs are defined as conjunctival inflammatory conditions associated with a type I allergy accompanied by some subjective and objective symptoms [3]. The Japanese Ocular Allergy Society conducted an online questionnaire to members of the Japan Ophthalmologists Association and their families in 2017 [30]. As a result, the prevalence of SAC, PAC, AKC, VKC, and GPC in Japan was reported to be 45.4%, 14.0%, 5.3%, 1.2%, and 0.6%, respectively. The morbidity of SAC was observed in children and was found to gradually increase with age. The morbidity of PAC peaked bimodally between the ages of 10 and 19 years, and 40 and 49 years, and AKC was bimodal in slightly younger generations. The morbidity of VKC was most prominent in people aged between 20 and 29 years. In the Japanese geographical distribution of SAC and PAC, the capital and central regions showed a high prevalence of SAC (especially caused by cedar/cypress pollen), whereas the distribution of PAC cases showed a marked geographical difference. Overall, therefore, the ACDs are estimated to occur in patients at all ages (especially at a young age) and in all regions. 

In Japan, allergic conjunctivitis is also screened at school checkups. However, the current situation is that it is difficult to make an accurate diagnosis and understand the progress. Moreover, it is often found that eye drops are initiated by non-ophthalmologists without an eye examination, with only subjective symptoms and a medical interview. Thus, as we mentioned, we believe that the SEC, which can continuously reconfirm images taken by moving images along the time axis, will play an active role. The average recording time in this study was about 30 s in both eyes, as in our previous report [22], suggesting that the SEC is easy to use even when healthcare workers, including physicians, are busy. The non-specialists can use this portable and recordable slit-lamp device, but an ophthalmologist must evaluate the recorded images to make a diagnosis.

SEC is a smartphone attachment that allows video recording [21], and thus, we believe that it may be useful in telemedicine in the near future. Additionally, there are some reports that the high prevalence of the severe ACDs is significantly associated with the levels of the air pollutants [30,31], which indicates that clinicians should be aware that air pollutants may cause serious risks leading to severe ocular allergic changes such as impaired vision due to epithelial keratopathy. Therefore, if Internet of Things (IoT) devices such as the SEC (small-sized medical device of smart phone attachment) can be used by not only ophthalmologists but also non-ophthalmologists to understand environmental factors, and if location information can be obtained, it can be applied to epidemiological surveys in the future.

The current study had several limitations. First, the number of participants was low. Moreover, there were differences in the number of different types of ACDs. Because the score was 0 points for GPC in palpebral conjunctiva, swelling, and Trantas dot in limbus, it was not confirmed in this study whether these findings could be accurately assessed by the SEC in this study. In addition, the correlation between the two devices may be high due to the small number of participants. However, in actual clinical practice, there are cases that are evaluated using SEC (Figures S1 and S2). We will increase the number of cases in the future. Second, the evaluation of corneal findings using fluorescein staining was difficult. Shield ulcers are expected to beigu definitively diagnosed, but when any one superficial punctate keratitis (SPK) is observed, the eye is defined as severe ACDs, which can influence clinical practice. The utility of SEC in mice in the dry eye model has been previously reported [21], but it should also be applied to diseases where corneal involvement (e.g., dry eye, infection, trauma) is diagnostic in humans [32]. Therefore, we will continue to evaluate the efficacy and safety of SEC in other anterior ocular diseases. 

## 5. Conclusions

The results of this study suggested that not only the conventional, non-portable slit-lamp microscope, but also the portable and recordable slit-lamp device, can diagnose and record allergic ocular images relatively accurately.

## 6. Patents

OUI, Inc. has the patent for the Smart Eye Camera (Japanese Patent No. 6627071. Inventors: H.Y., E.S., and N.A., Tokyo, Japan). There are no other relevant declarations relating to this patent.

## Figures and Tables

**Table 1:** Demographics of the subjects.

Cases	17	
Male/Female	12/5	
Age (y)	21.5 ± 14.8	
Eyes	34	
Perennial Allergic Conjunctivitis (PAC)	4	
Seasonal Allergic Conjunctivitis (SAC)	18	
Giant Papillary Conjunctivitis (GPC)	2	
Atopic Keratoconjunctivitis (AKC)	5	
Vernal Keratoconjunctivitis (VKC)	3	
Unclear or Lack of Images	2	
Average Scores (point)		
RE (Conventional/SEC)	3.3 ± 2.4/3.2 ± 2.1	0.33 *
LE (Conventional/SEC)	3.4 ± 2.4/4.3 ± 3.1	0.34 *
BE (Conventional/SEC)	6.6 ± 4.8/7.5 ± 3.6	0.43 *

Data Shown as Mean ± SD, * *p* value; Paired T-test, ACDs: Allergic Conjunctival Diseases, SEC: Smart Eye Camera, RE: Right Eye, LE: Left Eye, BE: Both Eyes.

**Table 2:** Reproducibility of the ACD scores evaluated by the two devices.

	Eye	Palpebral Conjunctiva					Bulbar Conjunctiva		Limbus		Cornea	Total
Sign		Hyperemia	Swelling	Follicle	Papillae	Giant Papillae	Hyperemia	Chemosis	Swelling	Trantas	Epithelial Disorder	
RE	***n***	17										
	***κ***	0.78	0.88	0.87	0.88	-	1.00	1.00	-	-	0.63	0.73
	95% CI	0.70–0.85	0.83–0.94	0.80–0.93	0.83–0.94	-	-	-	-	-	0.46–0.81	0.67–0.78
LE	***n***	17										
	***κ***	0.78	0.50	0.87	0.87	-	0.81	1.00	-	-	0.73	0.45
	95% CI	0.71–0.85	0.41–0.59	0.81–0.93	0.72–0.86	-	0.75–0.87	-	-	-	0.65–0.82	0.39–0.51
BE	***n***	34										
	***κ***	0.78	0.87	0.75	0.83	-	0.89	1.00	-	-	0.72	0.63
	95% CI	0.75–0.82	0.83–0.91	0.71–0.79	0.80–0.87	-	0.86–0.91	-	-	-	0.66–0.77	0.60–0.66

RE: Right Eye, LE: Left Eye, BE: Both Eyes, CI: confidence interval.

## Data Availability

All data associated with this study including raw data can be found on the data server in the Department of Ophthalmology, Tsurumi University School of Dental Medicine and Keio University School of Medicine.

## Supplementary Materials

The following are available online at https://www.mdpi.com/2075-4418/11/3/535/s1, Figure S1: Representative photographs of giant papillae in the palpebral conjunctiva by the SEC, Figure S2: Representative photographs of Trantas dots and swelling in the limbus by the SEC, Table S1: Grading scores of ten clinical signs, Table S2: Correlation of the severity scores of ACDs evaluated by the two devices.

## References

[1] Okuda M. (2003). Epidemiology of Japanese cedar pollinosis throughout Japan. Ann. Allergy Asthma Immunol..

[2] Okubo K., Kurono Y., Fujieda S., Ogino S., Uchio E., Odajima H., Takenaka H., Baba K. (2011). Japanese Society of A: Japanese guideline for allergic rhinitis. Allergol. Int..

[3] Miyazaki D., Takamura E., Uchio E., Ebihara N., Ohno S., Ohashi Y., Okamoto S., Satake Y., Shoji J., Namba K. (2020). Japanese guidelines for allergic conjunctival diseases 2020. Allergol. Int..

[4] Leonardi A., Motterle L., Bortolotti M. (2008). Allergy and the eye. Clin. Exp. Immunol..

[5] Fujishima H., Okada N., Matsumoto K., Fukagawa K., Igarashi A., Matsuda A., Ono J., Ohta S., Mukai H., Yoshikawa M. (2016). The usefulness of measuring tear periostin for the diagnosis and management of ocular allergic diseases. J. Allergy Clin. Immunol..

[6] Ibrahim O.M.A., Dogru M., Kaido M., Kojima T., Fujishima H., Tsubota K. (2014). Functional Visual Acuity Assessment of Severe Atopic Keratoconjunctivitis. Cornea.

[7] Sy H., Bielory L. (2013). Atopic keratoconjunctivitis. Allergy Asthma Proc..

[8] Fukushima A., Ohashi Y., Ebihara N., Uchio E., Okamoto S., Kumagai N., Shoji J., Takamura E., Nakagawa Y., Namba K. (2014). Therapeutic effects of 0.1% tacrolimus eye drops for refractory allergic ocular diseases with proliferative lesion or corneal involvement. Br. J. Ophthalmol..

[9] Yucel O.E., Ulus N.D. (2016). Efficacy and safety of topical cyclosporine A 0.05% in vernal keratoconjunctivitis. Singapore Med. J..

[10] Kumar S. (2009). Vernal keratoconjunctivitis: A major review. Acta Ophthalmol..

[11] Vichyanond P., Pacharn P., Pleyer U., Leonardi A. (2014). Vernal keratoconjunctivitis: A severe allergic eye disease with remodeling changes. Pediatr. Allergy Immunol..

[12] Yazu H., Fukagawa K., Shimizu E., Sato Y., Fujishima H. (2021). Long-term outcomes of 0.1% tacrolimus eye drops in eyes with severe allergic conjunctival diseases. Allergy Asthma Clin. Immunol..

[13] Zhong X., Liu H., Pu A., Xia X., Zhou X. (2007). M cells are involved in pathogenesis of human contact lens-associated giant papillary conjunctivitis. Arch. Immunol. Exp..

[14] Donshik P.C., Ehlers W.H., Ballow M. (2008). Giant Papillary Conjunctivitis. Immunol. Allergy Clin. N. Am..

[15] Zepeda Ortega B., Rosas Vargas M.A., Ito Tsuchiya F.M., del Rio Navarro B.E., Sienra Monge J.J. (2007). Allergic conjunctivitis in children. Rev. Alerg. Mexico.

[16] Fujishima H., Fukagawa K., Takano Y., Okamoto S., Nakagawa Y., Uchio E., Yokoi N., Fukushima A., Takamura E. (2006). The Early Efficacy of Topical Levocabastine in Patients with Allergic Conjunctivitis. Allergol. Int..

[17] O’Sullivan E.P., Malhotra R., Migdal C. (2001). Prescription of eye drops. Postgrad. Med. J..

[18] Al Hanaineh A.T., Hassanein D.H., Abdelbaky S.H., El Zawahry O.M. (2018). Steroid-induced ocular hypertension in the pediatric age group. Eur. J. Ophthalmol..

[19] Maier P., Lapp T., Reinhard T. (2017). Ocular involvement in atopic dermatitis: Clinical aspects and therapy. Ophthalmologe.

[20] Kolbe L., Kligman A.M., Schreiner V., Stoudemayer T. (2001). Corticosteroid-induced atrophy and barrier impairment measured by non-invasive methods in human skin. Ski. Res. Technol..

[21] Shimizu E., Ogawa Y., Yazu H., Aketa N., Yang F., Yamane M., Sato Y., Kawakami Y., Tsubota K. (2019). Smart Eye Camera: An innovative technique to evaluate tear film breakup time in a murine dry eye disease model. PLoS ONE.

[22] Yazu H., Shimizu E., Okuyama S., Katahira T., Aketa N., Yokoiwa R., Sato Y., Ogawa Y., Fujishima H. (2020). Evaluation of Nuclear Cataract with Smartphone-Attachable Slit-Lamp Device. Diagnostics.

[23] Shimizu E., Yazu H., Aketa N., Yokoiwa R., Sato S., Yajima J., Katayama T., Sato R., Tanji M., Sato Y. (2021). A Study Validating the Estimation of Anterior Chamber Depth and A Study Validating the Estimation of Anterior Chamber Depth and Iridocorneal Angle with Portable and Non-Portable Slit-Lamp Microscopy. Sensors.

[24] Yazu H., Shimizu E., Aketa N., Dogru M., Okada N., Fukagawa K., Fujishima H. (2019). The efficacy of 0.1% tacrolimus ophthalmic suspension in the treatment of severe atopic keratoconjunctivitis. Ann. Allergy Asthma Immunol..

[25] Takamura E., Uchio E., Ebihara N., Ohno S., Ohashi Y., Okamoto S., Kumagai N., Satake Y., Shoji J., Nakagawa Y. (2017). Japanese guidelines for allergic conjunctival diseases 2017. Allergol. Int..

[26] Gloor B.R. (2010). Hans Goldmann (1899–1991). Eur. J. Ophthalmol..

[27] Chen D.Z., Tan C.W. (2016). Smartphone Imaging in Ophthalmology: A Comparison with Traditional Methods on the Reproducibility and Usability for Anterior Segment Imaging. Ann. Acad. Med. Singap..

[28] Dubbs S.B., Blosser K.M., Richardson A.C. (2019). A smartphone, a slit lamp, and an ophthalmology consult. Clin. Case Rep..

[29] Mohammadpour M., Mohammadpour L., Hassanzad M. (2016). Smartphone Assisted Slit Lamp Free Anterior Segment Imaging: A novel technique in teleophthalmology. Contact Lens Anterior Eye.

[30] Miyazaki D., Fukagawa K., Fukushima A., Fujishima H., Uchio E., Ebihara N., Shoji J., Takamura E., Namba K., Ohashi Y. (2019). Air pollution significantly associated with severe ocular allergic inflammatory diseases. Sci. Rep..

[31] Mimura T., Ichinose T., Yamagami S., Fujishima H., Kamei Y., Goto M., Takada S., Matsubara M. (2014). Airborne particulate matter (PM2.5) and the prevalence of allergic conjunctivitis in Japan. Sci. Total Environ..

[32] Shimizu E., Yazu H., Aketa N., Yokoiwa R., Sato S., Katayama T., Hanyuda A., Sato Y., Ogawa Y., Tsubota K. (2021). Smart Eye Camera: A validation study for evaluating the tear film breakup time in human subjects. Transl. Vis. Sci. Technol..

