# Supplementary material for: Clinical Observation of Allergic Conjunctival Diseases with Portable and Recordable Slit-Lamp Device
Source: Diagnostics (Basel). 2021 Mar 17;11(3):535. doi: 10.3390/diagnostics11030535 (PMC8002473; doi:10.3390/diagnostics11030535)
Supplement: Supplementary file 1 [file diagnostics-11-00535-s001.zip › Supplementary files/Figure S2.docx]

**Figure S2** Representative photographs of Trantas dots and swelling in the limbus by the SEC


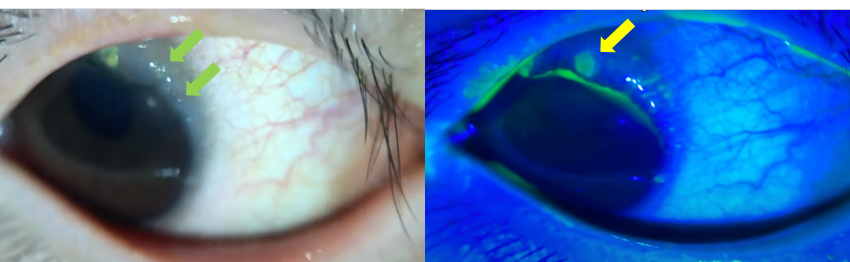


(a) (b)

(a) An 11-year-old male patient was diagnosed with severe AKC with Trantas dots and swelling in the nasal limbus (green arrows). (b) Findings after fluorescein staining. Note the epithelial defect on the Trantas dots (yellow arrow).
