# Supplementary material for: Clinical Observation of Allergic Conjunctival Diseases with Portable and Recordable Slit-Lamp Device
Source: Diagnostics (Basel). 2021 Mar 17;11(3):535. doi: 10.3390/diagnostics11030535 (PMC8002473; doi:10.3390/diagnostics11030535)
Supplement: Supplementary file 1 [file diagnostics-11-00535-s001.zip › Supplementary files/Table S2.docx]

**Table S2** Correlation of the severity scores of ACDs evaluated by the two devices*.*

| Eye Sign | | palpebral conjunctiva | | | | | bulbar conjunctiva | | limbus | | cornea | total |
| --- | --- | --- | --- | --- | --- | --- | --- | --- | --- | --- | --- | --- |
|  |  | hyperemia | swelling | follicle | papillae | giant papillae | hyperemia | chemosis | swelling | trantas | epithelial disorder |  |
| RE | **n** | 17 | | | | | | | | | | |
|  | **r** | 0.91 | 0.90 | 0.87 | 0.91 | - | 1.00 | 1.00 | - | - | 0.68 | 0.95 |
|  | **95% CI** | 0.75-0.97 | 0.73-0.97 | 0.64-0.96 | 0.74-0.97 | - | - | - | - | - | 0.20-0.89 | 0.86-0.98 |
| LE | **n** | 17 | | | | | | | | | | |
|  | **r** | 0.93 | 0.92 | 0.66 | 0.88 | - | 0.89 | 1.00 | - | - | 0.78 | 0.88 |
|  | **95% CI** | 0.81-0.98 | 0.76-0.97 | 0.24-0.87 | 0.69-0.96 | - | 0.70-0.96 | - | - | - | 0.47-0.92 | 0.69-0.96 |
| BE | **n** | 34 | | | | | | | | | | |
|  | **r** | 0.90 | 0.87 | 0.74 | 0.93 | - | 0.94 | 1.00 | - | - | 0.73 | 0.92 |
|  | **95% CI** | 0.80-0.95 | 0.75-0.94 | 0.53-0.87 | 0.85-0.96 | - | 0.87-0.97 | - | - | - | 0.51-0.86 | 0.84-0.96 |

RE: Right Eye, LE: Left Eye, BE: Both Eyes, CI: confidence interval

*Spearman’s rank correlation coefficient
