# Supplementary material for: Clinical Observation of Allergic Conjunctival Diseases with Portable and Recordable Slit-Lamp Device
Source: Diagnostics (Basel). 2021 Mar 17;11(3):535. doi: 10.3390/diagnostics11030535 (PMC8002473; doi:10.3390/diagnostics11030535)
Supplement: Supplementary file 1 [file diagnostics-11-00535-s001.zip › Supplementary files/Figure S1.docx]

**Figure S1** Representative photographs of giant papillae by the SEC.


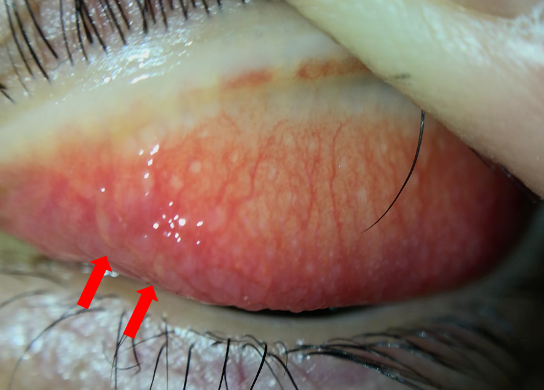


An 14-year-old male patient was diagnosed with severe AKC with giant papillae that occupied the nasal palpebral conjunctiva in his left eye (red arrows).
