# Supplementary material for: Clinical Observation of Allergic Conjunctival Diseases with Portable and Recordable Slit-Lamp Device
Source: Diagnostics (Basel). 2021 Mar 17;11(3):535. doi: 10.3390/diagnostics11030535 (PMC8002473; doi:10.3390/diagnostics11030535)
Supplement: Supplementary file 1 [file diagnostics-11-00535-s001.zip › Supplementary files/Table S1 .docx]

**Table S1** Grading scores of ten clinical signs.
